# Forty-Year Trends in Tooth Loss Among American Adults With and Without Diabetes Mellitus: An Age-Period-Cohort Analysis

**DOI:** 10.5888/pcd12.150309

**Published:** 2015-12-03

**Authors:** Huabin Luo, Wei Pan, Frank Sloan, Mark Feinglos, Bei Wu

**Affiliations:** Author Affiliations: Huabin Luo, Brody School of Medicine, East Carolina University, Greenville, North Carolina; Wei Pan, Frank Sloan, Mark Feinglos, Duke University, Durham, North Carolina.

## Abstract

**Introduction:**

This study aimed to assess the trends in tooth loss among adults with and without diabetes mellitus in the United States and racial/ethnic disparities in tooth loss patterns, and to evaluate trends in tooth loss by age, birth cohorts, and survey periods.

**Methods:**

Data came from 9 waves of the National Health and Nutrition Examination Survey (NHANES) from 1971 through 2012. The trends in the estimated tooth loss in people with and without diabetes were assessed by age groups, survey periods, and birth cohorts. The analytical sample was 37,609 dentate (ie, with at least 1 permanent tooth) adults aged 25 years or older. We applied hierarchical age-period-cohort cross-classified random-effects models for the trend analysis.

**Results:**

The estimated number of teeth lost among non-Hispanic blacks with diabetes increased more with age than that among non-Hispanic whites with diabetes (*z* = 4.05, *P* < .001) or Mexican Americans with diabetes (*z* = 4.38, *P* < .001). During 1971–2012, there was a significant decreasing trend in the number of teeth lost among non-Hispanic whites with diabetes (slope = −0.20, *P* < .001) and non-Hispanic blacks with diabetes (slope = −0.37, *P* < .001). However, adults with diabetes had about twice the tooth loss as did those without diabetes.

**Conclusion:**

Substantial differences in tooth loss between adults with and without diabetes and across racial/ethnic groups persisted over time. Appropriate dental care and tooth retention need to be further promoted among adults with diabetes.

## Introduction

The prevalence of diabetes has increased rapidly in the United States since the mid-1990s (1). In 2012, an estimated 28.9 million people aged 20 years or older had diabetes, and the prevalence is higher in racial/ethnic minority groups: 13.2% in non-Hispanic blacks, and 12.8% in Hispanics, compared with 7.6% in non-Hispanic whites (2). Research shows a bidirectional relationship between diabetes and periodontal disease (3). Periodontal disease is considered the sixth complication of diabetes (4) and has been identified as a risk factor for poor metabolic control in people with diabetes (3). About half of US adults have periodontal disease (5), and the prevalence of periodontal disease is even higher for adults with diabetes (6). Further, periodontal disease is a major risk factor for tooth loss (7).

Although many studies have examined the trend in tooth loss for the general population (8,9), population-based studies on tooth loss in adults with diabetes in the United States are rare (10). There have been significant improvements in awareness of chronic disease and technological and pharmacological treatment options for diabetes (eg, use of metformin) and oral health (eg, fluoride) (11). These developments may affect the relationship between diabetes and oral health. Thus, an evaluation of trends in tooth loss, a surrogate marker of oral health, in adults with diabetes is warranted. Our study will generate new knowledge for public health to support the Healthy People 2020 objective of reducing tooth loss (12).

Methodologically, most research on trends in oral health in the United States (13) has assumed that observed changes over survey years reflect period effects, after accounting for age effects. These studies did not account for birth cohort effects. Age, survey period, and birth cohort effects refer to time-related variations in the outcome of interest, but they have distinct meanings and are linearly dependent (cohort = period – age) (14). Age effects refer to variation associated with different age groups and reflect biological and social processes of aging internal to individuals and represent developmental changes over the life course. Period effects refer to changes in social, economic, technological, or physical environments affecting all age groups simultaneously at the time health is measured. Finally, cohort effects refer to variation among people in different birth cohorts. People in a cohort experience the same historical events (eg, the Great Depression). Thus, not taking into account cohort effects may lead to biased estimates of trends in social inequalities in the outcome of interest (14). A recent study (13) assessed the trend in complete tooth loss by treating the relationships between individual factors and the outcome of interest as homogenous across different survey periods and birth cohorts. Such an approach did not capture the dynamic biological and social processes and could generate biased estimates. Moreover, the study assessed trends only in the general US population.

To address these limitations, we examined trends in tooth loss among US adults with and without diabetes and across racial/ethnic groups in the National Health and Nutrition Examination Surveys (NHANES) 1971–2012, using age-period-cohort analysis (14) to account for potential random effects of survey period and birth cohort in addition to age effects. The study objectives were 1) to assess the trend in tooth loss and differences in trends in tooth loss for adults with and without diabetes and across racial/ethnic groups and 2) to evaluate the trends by age groups, birth cohorts, and survey periods.

## Methods

Data were obtained from 9 waves of national survey data: NHANES I (1971–1975), NHANES III (1988–1994), and 7 NHANES continuous surveys from 1999 to 2012. NHANES uses a multistage household probability sample from which noninstitutionalized US populations are selected, interviewed, and examined (15). In these surveys, 37,609 dentate individuals (ie, those with at least 1 permanent tooth) aged 25 or older received an oral examination. We excluded those with complete tooth loss and other racial/ethnic groups (ie, Asians, Native Americans, and Hispanics whose country of origin was not Mexico) because of insufficient sample size (in NHANES I and III) to allow valid population estimates.

The outcome variable was the number of teeth lost, which was determined by a dental professional during an oral health examination (15). Third molars were excluded from the counts presented here because they are typically removed by choice (16). Functional dentition was classified as having more than 21 teeth (17).

The presence of diabetes was determined by a self-reported response to the question “Have you ever been told by a doctor or health care professional that you have diabetes or sugar diabetes?” Responses included yes, no, or borderline. We classified participants with borderline diabetes as not having diabetes.

Demographic variables were age, sex, race, education, and poverty level. Adults older than 85 were coded as age >85 in NHANES III and NHANES 1999–2006, whereas the highest age in NHANES I was 74 and in NHANES 2007–2012 was 80. Age was centered at its mean (52 years). Race was coded as non-Hispanic white, non-Hispanic black, or Mexican American. Educational attainment was coded as less than high school, high school, or college or higher. Poverty level was assessed on the basis of the Poverty Index Ratio (PIR), the ratio of total family income to the US poverty level. We classified PIR into 4 quartiles. Finally, we created 17 birth cohorts from 1897–1904 to 1980–1986 with 5-year increments and 13 age groups from 25–29 years to 85 or older with 5-year increments.

**Figure 1:**
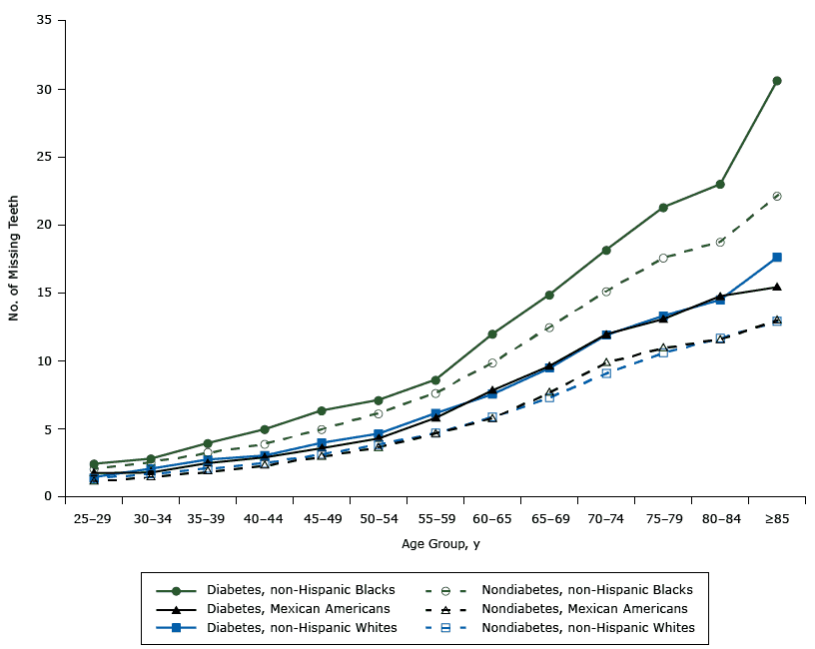
Estimated number of teeth lost by age groups. National Health and Nutrition Examination Survey (NHANES) 1971–2012. **Table:** 

**Figure 2:**
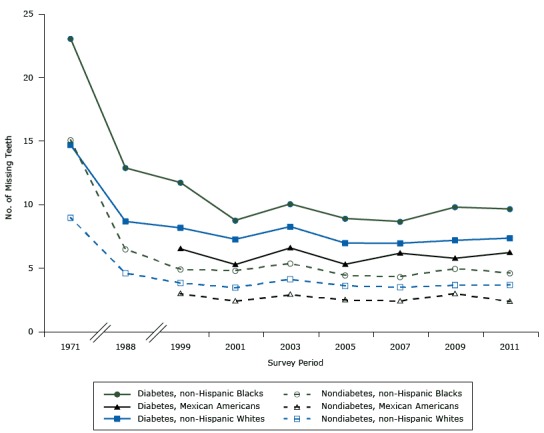
Estimated number of teeth lost by survey periods. National Health and Nutrition Examination (NHANES) 1971–2012. **Table:** 

**Figure 3:**
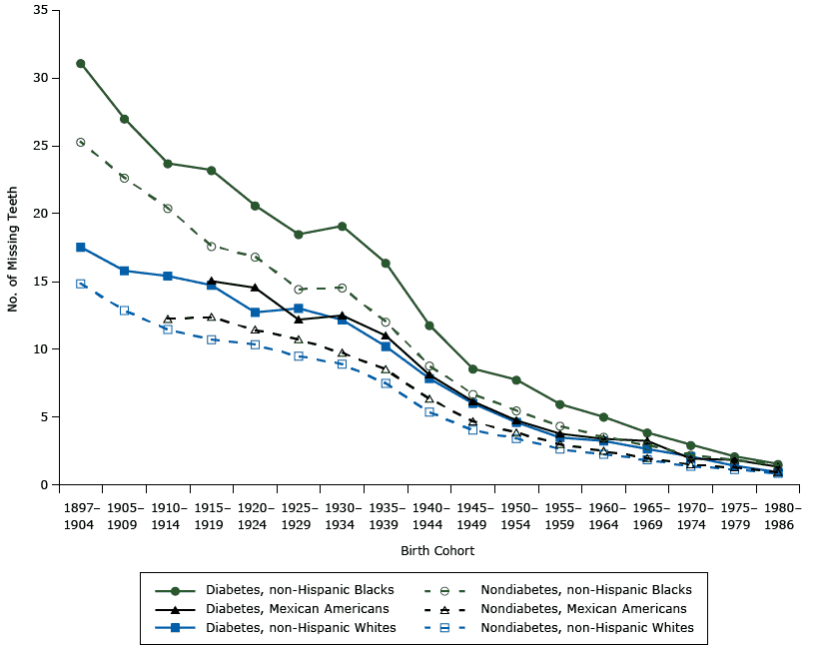
Estimated number of teeth lost by birth cohort. National Health and Nutrition Examination Survey (NHANES) 1971–2012. **Table:** 

We applied the hierarchical age-period-cohort (HAPC) cross-classified random-effects model (CCREM) (14) to adults with and without diabetes. Data analyses were conducted by using SAS PROC GLIMMIX (SAS Institute Inc) (18) with DIST = negative binomial because the number of missing teeth is an overdispersed count variable. HAPC-CCREM was developed for repeated cross-sectional surveys such as NHANES to deal with the clustering of data by survey periods and birth cohorts. This model can estimate any random clustering effects at higher-level cross-classified units such as survey periods and birth cohorts (19). We used clinical examination sampling weights in analyses. Given the large sample size, the criterion for statistical significance was set at *P* ≤ .01 or less. Trends in the estimated number of teeth lost in different age groups (Figure 1), survey periods (Figure 2), and birth cohorts (Figure 3) were profiled, and the slopes of trend were compared by diabetes status and race/ethnicity (20). We applied regression imputation to address missing values in the study sample.

Age Group, y	Adults With Diabetes, by Race/Ethnicity			Adults Without Diabetes, by Race/Ethnicity		
	White	Black	Mexican	White	Black	Mexican
25–29	1.46	2.44	1.75	1.38	2.06	1.13
30–34	2.08	2.82	1.79	1.70	2.56	1.44
35–39	2.74	3.96	2.49	2.09	3.22	1.83
40–44	3.04	4.98	2.91	2.48	3.90	2.29
45–49	3.98	6.36	3.58	3.14	4.97	2.95
50–54	4.66	7.14	4.29	3.85	6.16	3.65
55–59	6.16	8.62	5.80	4.69	7.65	4.66
60–65	7.56	11.99	7.84	5.86	9.87	5.78
65–69	9.46	14.88	9.62	7.31	12.48	7.66
70-–4	11.90	18.19	11.97	9.08	15.13	9.87
75–79	13.32	21.30	13.07	10.59	17.59	10.93
80–84	14.49	22.99	14.77	11.66	18.76	11.59
≥85	17.62	30.62	15.42	12.91	22.16	12.98

Survey Period	Adults With Diabetes, by Race			Adults Without Diabetes, by Race		
	White	Black	Mexican	White	Black	Mexican
1971	14.70	23.07	—	8.98	15.09	—
1988	8.68	12.89	—	4.61	6.51	—
1999	8.18	11.75	6.53	3.84	4.92	2.99
2001	7.28	8.78	5.29	3.48	4.82	2.40
2003	8.28	10.07	6.60	4.14	5.39	2.91
2005	6.98	8.92	5.31	3.61	4.44	2.50
2007	6.97	8.68	6.19	3.51	4.33	2.42
2009	7.20	9.81	5.79	3.66	4.98	2.98
2011	7.37	9.67	6.24	3.69	4.64	2.39

Birth Cohort	Adults With Diabetes, by Race			Adults Without Diabetes, by Race		
	White	Black	Mexican	White	Black	Mexican
1897–1904	17.54	31.11	—	14.84	25.30	—
1905–1909	15.80	27.02	—	12.86	22.65	—
1910–1914	15.41	23.71	—	11.45	20.43	12.24
1915–1919	14.73	23.23	15.03	10.73	17.62	12.37
1920–1924	12.74	20.62	14.55	10.34	16.85	11.43
1925–1929	13.03	18.49	12.20	9.50	14.45	10.73
1930–1934	12.18	19.09	12.50	8.91	14.56	9.74
1935–1939	10.22	16.37	11.01	7.50	12.04	8.54
1940–1944	7.85	11.79	8.12	5.38	8.81	6.36
1945–1949	6.02	8.58	6.17	4.04	6.72	4.67
1950–1954	4.63	7.76	4.76	3.41	5.50	3.88
1955–1959	3.50	5.96	3.78	2.65	4.37	3.00
1960–1964	3.25	5.02	3.41	2.26	3.55	2.50
1965–1969	2.65	3.87	3.25	1.86	2.93	1.99
1970–1974	2.10	2.98	1.95	1.38	2.23	1.52
1975–1979	1.43	2.12	1.85	1.15	1.88	1.28
1980–1986	0.95	1.56	1.35	0.87	1.46	0.92

## Results

Adults with diabetes were older than those without diabetes (*P* < .001) (Table 1). Over time, diabetes prevalence was consistently higher among non-Hispanic blacks and Mexican Americans than among non-Hispanic whites (*P* < .001). Adults with diabetes had lower income (*P* = .003) and lower educational attainment (*P* < .001), and more tooth loss than those without diabetes (except in NHANES I) (*P* < .001). From 1971 to 2012, the number of missing teeth decreased from 11.2 to 6.6 with a slope of −0.13 (*P* < .001) for the group with diabetes and from 9.4 to 3.4 with a slope of −0.16 (*P* < .001) in the group without diabetes (Table 2). The rate of decrease (slopes) between the 2 groups did not differ significantly (*P* = .36). An increasing trend was found for the rate of functional dentition for both groups. The rate for the group with diabetes increased from 38.6% in 1971 to 68.8% in 2012 with a slope of 0.81 (*P* < .001); the rate for the group without diabetes increased from 52.3% to 86.6% with a slope of 0.89 (*P* < .001). The rate of increase was not significantly different. 

**Table 1:** Descriptive Statistics of Respondents With and Without Diabetes (N = 37,609 Dentate People Aged ≥25), NHANES 1971–2002^a^

Variables	1971–1974 (n = 2,927)		1988–1994 (n = 11,978)		1999–2001 (n = 3,015)		2001–2002 (n = 3,453)	
	DM	No DM	DM	No DM	DM	No DM	DM	No DM
**Age, mean, y**	54.2	45.6	55.9	44.8	56.9	45.7	57.0	46.5
**Race/ethnicity**								
Non-Hispanic white	2.3	97.7	4.8	95.2	4.9	95.1	5.3	94.7
Non-Hispanic black	4.8	95.2	7.2	92.8	9.9	90.1	9.4	90.6
Mexican American	NA	NA	NA	NA	6.4	93.6	8.3	91.7
**Sex**								
Female	3.0	97.0	5.4	94.6	4.7	95.3	5.7	94.3
Male	2.1	97.9	4.7	95.3	6.4	93.6	6.3	93.7
**Poverty Index Ratio^b^**								
Quartile 1	5.3	94.7	7.5	92.5^a^	8.0	92.0	9.3	90.7
Quartile 2	2.3	97.7	7.1	92.9	8.9	91.1	8.9	91.1
Quartile 3	2.5	97.5	4.8	95.2	6.8	93.2	5.5	94.5
Quartile 4	1.6	98.4	3.9	96.1	3.1	96.9	4.5	95.5
**Education**								
Less than high school	5.5	94.5	11.2	88.8	9.9	90.1	9.6	90.4
High school	2.3	97.7	5.2	94.8	6.3	93.7	5.8	94.2
College and above	1.3	98.7	3.6	96.4	3.7	96.3	5.2	94.8
**Dental status**								
Missing teeth, mean, n	11.2	9.4	8.8	4.8	9.1	3.9	6.7	3.5
Functional dentition (≥21 teeth)	38.6	52.3	53.8	78.0	50.7	84.3	66.7	86.3

Abbreviations: DM, diabetes mellitus; NA, not included due to insufficient sample size; NHANES, National Health and Nutrition Examination Survey.

a Values are percentages unless otherwise indicated.

b The ratio of total family income to the US poverty level; 1st quartile (the lowest income) to 4th quartile (the highest income).

**Table 2:** Descriptive Statistics of Respondents With and Without Diabetes (N = 37,609 Dentate People Aged ≥25), NHANES 2003–2012^a^

Variables	2003–2004 (n = 3,193)		2005–2006 (n = 3,275)		2007–2008 (n = 3,611)		2009–2010 (n = 3,176)		2011–2012 (n = 2,981)	
	DM	No DM	DM	No DM	DM	No DM	DM	No DM	DM	No DM
**Mean age, y**	58.4	46.9	57.3	47.6	57.4	47.8	59.8	50.1	59.6	48.7
**Race/ethnicity**										
Non-Hispanic white	6.3	93.7	6.2	93.8	7.0	93.0	7.2	92.8	8.3	91.7
Non-Hispanic black	11.3	88.7	12.1	87.9	14.9	85.1	13.4	86.6	14.3	85.7
Mexican American	7.9	92.1	8.7	91.3	8.2	91.8	10.5	89.5	10.3	89.7
**Sex**										
Female	7.0	93.0	7.5	92.5	7.9	92.1	7.4	92.6	9.0	91.0
Male	7.0	93.0	6.7	93.3	8.2	91.8	9.2	90.8	9.5	90.5
**Poverty Index Ratio^b^**										
Quartile 1	8.4	91.6	8.9	91.1	9.3	90.7	9.9	90.1	12.4	87.6
Quartile 2	8.1	91.9	9.4	90.6	11.5	88.5	11.4	88.6	12.7	87.3
Quartile 3	8.6	91.4	8.4	91.6	9.1	90.9	9.9	90.1	10.4	89.6
Quartile 4	5.4	94.6	4.8	95.2	5.8	94.2	5.8	94.2	6.4	93.6
**Education**										
Less than high school	10.6	89.4	10.4	89.6	13.3	86.7	11.4	88.5	13.6	86.4
High school	7.4	92.6	7.6	92.4	8.7	91.3	8.6	91.4	12.1	87.9
College and above	6.0	94.0	6.1	93.9	6.3	93.7	7.4	92.6	7.4	92.6
**Dental status**										
Missing teeth, mean, n	8.5	4.0	6.2	3.5	6.9	3.4	7.2	3.6	6.6	3.4
Functional dentition (≥21 teeth)	56.6	82.1	67.4	85.2	65.3	86.1	65.7	84.9	68.8	86.6

Abbreviations: DM, diabetes mellitus; NHANES, National Health and Nutrition Examination Survey.

a Values are percentages unless otherwise indicated.

b The ratio of total family income to the US poverty level; 1st quartile (the lowest income) to 4th quartile (the highest income).

HAPC-CCREM model results (Table 3) demonstrate that having diabetes was a significant risk factor for tooth loss (B = 0.18,* P* < .001). Compared with non-Hispanic whites, non-Hispanic blacks were at higher risk for tooth loss (B = 0.29,* P* < .001), although Mexican Americans were at lower risk (B = −0.27,* P* < .001). Parameter estimates on all other covariates were significant (*P* < .001) except for sex.

**Table 3:** Hierarchical Age-Period-Cohort Cross-Classified Model Results (N = 37,609 Dentate People Aged ≥25), NHANES 1971–2012

Variable	Dependent Variable	
	Missing Teeth	Functional Dentition
	Estimate (SE)	
**Fixed effects**		
Intercept	1.96 (0.12)	0.02 (0.14)
Diabetes	0.18 (0.03)	−0.43 (0.01)
Age (centered)	0.04 (0.002)	−0.07 (0)
Female	0.01 (0.015)	0.04 (0)
**Race/ethnicity**		
Non-Hispanic white	1 [Reference]	
Non-Hispanic black	0.29 (0.02)	−0.38 (0.01)
Mexican American	−0.27 (0.04)	0.87 (0.01)
**Education**		
Less than high school	1 [Reference]	
High school	−0.07 (0.02)	0.38 (0)
College and above	−0.52 (0.02)	1.31 (0)
**Poverty Index Ratio^a^**		
Quartile 1	1 [Reference]	
Quartile 2	−0.05 (0.03)	0.09 (0.01)
Quartile 3	−0.52 (0.02)	1.31 (0.01)
Quartile 4	−0.45 (0.03)	0.98 (0.01)
**Random effects**		
Survey period	0.10 (0.05)	0.13 (0.06)
1971–1974	0.75 (0.12)	−0.71 (0.12)
1988–1994	0.06 (0.11)	−0.36 (0.12)
1999–2000	−0.12 (0.11)	−0.01 (0.12)
2001–2002	−0.22 (0.11)	0.21 (0.12)
2003–2004	−0.07 (0.11)	−0.18 (0.12)
2005–2006	−0.25 (0.11)	0.23 (0.12)
2007–2008	−0.28 (0.11)	0.23 (0.12)
2009–2010	−0.24 (0.12)	0.25 (0.12)
2011–2012	−0.20 (0.12)	0.34 (0.12)
**Birth cohort**	0.04 (0.01)	0.12 (0.04)
1897–1904	−0.29 (0.10)	−0.02 (0.08)
1905–1909	−0.17 (0.09)	−0.01 (0.08)
1910–1914	−0.10 (0.08)	−0.09 (0.08)
1915–1919	−0.03 (0.07)	−0.15 (0.08)
1920–1924	0.05 (0.07)	−0.06 (0.08)
1925–1929	0.11 (0.06)	−0.23 (0.08)
1930–1934	0.22 (0.06)	−0.38 (0.08)
1935–1939	0.29 (0.05)	−0.58 (0.08)
1940–1944	0.21 (0.05)	−0.33 (0.08)
1945–1949	0.14 (0.05)	−0.24 (0.08)
1950–1954	0.12 (0.06)	−0.09 (0.08)
1955–1959	0.03 (0.06)	0.08 (0.08)
1960–1964	0 (0.06)	0.15 (0.08)
1965–1969	−0.06 (0.07)	0.29 (0.08)
1970–1974	−0.15 (0.08)	0.50 (0.08)
1975–1979	−0.21 (0.09)	0.40 (0.08)
1980–1986	−0.36 (0.11)	0.76 (0.08)

Abbreviations: NHANES, National Health and Nutrition Examination Survey; SE, standard error.

a The ratio of total family income to the US poverty level; 1st quartile (the lowest income) to 4th quartile (the highest income).

Overall, birth cohort had a significant random effect on tooth loss (B = 0.04,* P* = .005); 6 of the 17 random cohort effects were significant. For example, people born in 1897–1904 (the oldest cohort) and 1980–1986 (the youngest cohort) had significantly slower tooth loss than people born in other birth cohorts, whereas people born in 1930–1949 (during the Great Depression) had significantly faster tooth loss than people born in other birth cohorts, which caused a “bump” for birth cohorts from 1930–1934 and 1945–1949, especially for non-Hispanic blacks, indicating birth cohort effects (see Figure 3). The overall random period effect was, however, not significant (Table 3).

Tooth loss increased with age group (Figure 1), but the rate of increase varied by diabetes status and race/ethnicity. In the group with diabetes, tooth loss increased faster in non-Hispanic blacks than in non-Hispanic whites (*z* = 4.05, *P* < .001) or in Mexican Americans (*z* = 4.38, *P* < .001). In the group without diabetes, tooth loss also increased faster in non-Hispanic blacks than in non-Hispanic whites (*z* = 5.10, *P* < .001) or in Mexican Americans (*z* = 4.73, *P* < .001). By racial/ethnic group, tooth loss increased faster with age among non-Hispanic whites with diabetes than among their counterparts without diabetes (*z* = 2.73, *P* = .01). However, the differences in tooth loss between non-Hispanic blacks with and without diabetes or between Mexican Americans with and without diabetes were not significant.

From 1971 to 2012, overall, a decreasing trend was seen in the number of teeth lost among non-Hispanic whites with diabetes (slope = −0.20, *P* < .001) and without diabetes (slope = −0.14, *P* < .001); and among non-Hispanic blacks with (slope = −0.37, *P* < .001) and without diabetes (slope = −0.27, *P* < .001) (Figure 2). However, from 1999 to 2012, no significant decreasing trend was found for Mexican Americans with and without diabetes.

In addition, the within- and between-group comparisons for the period 1971–2012 show that the slopes of tooth loss were not significant. We did not compare Mexican Americans with the other 2 racial groups because Mexican Americans were not sufficiently sampled in NHANES I and III. Nonetheless, we saw 3 consistent patterns (Figure 2). Having diabetes consistently led to more tooth loss regardless of race/ethnicity. On average, adults with diabetes lost approximately twice the number of teeth as adults without diabetes, ranging from 1.7 in 1971–1975 to 2.1 times in 2011–2012. Non-Hispanic blacks lost the greatest number of teeth, followed by non-Hispanic whites and Mexican Americans. Rates of tooth loss did not change significantly from 1999 onward, regardless of diabetes status or race/ethnicity (Figure 2).

Members of younger birth cohorts had significantly fewer missing teeth than members of older cohorts (Figure 3). The trend in tooth loss for some cohorts (eg, 1930–1934) exhibited larger changes. In particular, cohort effects were more pronounced for non-Hispanic blacks.

We conducted additional analyses for functional dentition by using a HAPC-CCREM model (Table 2, 4th and 5th columns) and assessed its trend from 1971 to 2012. Results are similar to those using the outcome as number of missing teeth. The rate of functional dentition decreased with age. It was lower among adults with diabetes than among adults without the disease, ranging from 14% lower in 1971–1975 to 34% lower in 1999–2000. Non-Hispanic blacks with diabetes had the lowest rate of functional dentition.

## Discussion

To our knowledge, this is the first study to assess trends in tooth loss among dentate adults with diabetes from 1971 to 2012, accounting for age, survey period, and cohort effects. We found a significant decline in tooth loss among adults with diabetes, similar to that found in adults without diabetes. Furthermore, tooth loss declined more for adults with diabetes from 1971 through 2012, although it did not differ significantly from the decline seen for adults without diabetes.

Our study showed that the trends in tooth loss for adults with and without diabetes were similar for the 3 racial/ethnic groups; however, the gaps between adults with and without diabetes remained. These findings have important implications. Other investigators have reported that tooth loss is associated with lower consumption of dietary fiber, fruits and vegetables, and a higher intake of cholesterol and saturated fatty foods (21). Such a diet is the opposite of what is recommended for patients with diabetes. Moreover, increasing evidence suggests a relationship between oral health and other chronic diseases, including cardiovascular diseases, respiratory diseases, and cognitive decline (22,23). Thus, the importance of tooth retention in adults needs to be emphasized to both patients and health care providers.

Our results are consistent with prior research in the general population over a shorter time span (8,9). Dye and colleagues reported the number of missing teeth decreased for all adult groups from 1988–1994 to 1999–2004 (9). The HAPC-CCREM model results showed that diabetes remained a significant risk factor for tooth loss among dentate adults aged 25 or older, which is consistent with prior studies (10,24). Among people aged 18 or older, Kapp and colleagues found that adults with diabetes had 1.5 times the odds of having at least one tooth removed as adults of the same age without diabetes (24). Moreover, our study results show that Mexican Americans are at lower risk of tooth loss than are non-Hispanic whites. A prior study also reported that Mexican Americans aged 50 or older had lost fewer teeth than their white counterparts (8). However, research that provides a historical context of dental care and the attitude of the culture toward tooth retention is limited. Future research is needed to provide more insight into the study findings.

The study results show significant birth cohort effects. That is, members of the same birth cohorts experienced the same historical events, including social and economic changes and technological developments since birth. These circumstances affected tooth loss. For example, there are 2 “bump points” in the trend line of tooth loss for non-Hispanic blacks with diabetes born during 1930–1934 and 1935–1939 (Figure 3), indicating more tooth loss for these 2 birth cohorts than for cohorts immediately before or after them. This finding may suggest the impact of common historical events (eg, the Great Depression) at birth.

The persistently lower rate of dental care in non-Hispanic blacks in the past (25) could account for the greater tooth loss in older black birth cohorts found in our study. Historically, non-Hispanic blacks particularly faced a challenge obtaining proper dental care because of a lack of dental services and dental knowledge (26). An earlier study found that in 1977, 23.4% of non-Hispanic blacks visited a dentist in the past year, compared with 46.9% of whites and 29.9% of Hispanics (25).

Moreover, regardless of diabetes status or race/ethnicity, the differences in slopes diverged more for older cohorts. Better access to dental care, more awareness and knowledge of oral health, technological change, and improvements in dental hygiene practice (11) could account for the convergence in trends in tooth loss (ie, smaller differences) in younger cohorts. Finally, after age and cohort effects were accounted for, the survey period effect was not significant.

Our findings on trends in tooth loss provide important information for future oral health policy and intervention programs. We found that trend lines in the number of teeth lost diverged more after age 60 among the 3 racial/ethnic groups. In addition, the rate of tooth loss increased more with age among non-Hispanic blacks than among non-Hispanic whites and Mexican Americans; and non-Hispanic blacks with diabetes had the fastest increase in rate of tooth loss. These findings indicate the cumulative effects of aging on tooth loss, especially among non-Hispanic blacks with diabetes. Targeted initiatives are needed to improve health literacy in people with diabetes, particularly racial/ethnic minority populations. Improved access to dental care is essential for the care of adults with diabetes. Prior research shows that periodontal diseases are more prevalent and more severe in adults with diabetes than those without diabetes (6). Yet, a prior study reported that adults with diabetes were less likely to have seen a dentist than those without diabetes in the past 12 months (65.8% vs 73.1%) (27). Thus, regular dental service should become an integral part of diabetes disease management.

Another factor accounting for the greater tooth loss among adults with diabetes is poor oral hygiene. Research has demonstrated that people with diabetes have poorer oral health-related behaviors. They do not brush and floss as often as people without diabetes (28). Given the bidirectional relationship between diabetes and periodontal disease, our study findings highlight the need to improve dental self-care and knowledge of diabetes risks among people with diabetes, especially among non-Hispanic blacks, who had more tooth loss and lost teeth at a higher rate.

We acknowledge several study limitations. The cross-sectional design did not account for temporality — that is, whether onset of diabetes preceded tooth loss in these people. Diabetes status was self-reported; thus, it raises concern for validity. Nonetheless, self-reported diabetes status strongly agrees with medical record data (29). Additionally, there were few observations for Mexican Americans in NHANES I and III, which limited the trend comparison by race/ethnicity before 1999.

Our study found that substantial differences in tooth loss between adults with and without diabetes have persisted over time. Adults with diabetes lost more teeth than adults without the disease. Non-Hispanic blacks with diabetes lost the largest number of teeth, and they had the greatest increase in tooth loss as they aged. The importance of necessary dental care and tooth retention needs to be further promoted among patients and clinical providers. Growing evidence shows that oral health is associated with general health (22). To control diabetes complications, an interprofessional and team-based approach is needed to ensure better care coordination and disease management (30).
